# Medical Items

**Published:** 1894-11

**Authors:** 


### *MEDICAL ITEMS.*

---

Dr. Bernard Wolff and Miss Marion Hillyer, both of Atlanta, were married on the evening of October 18th. The Journal offers congratulations and best wishes for a long life of happiness and success.

---

The twentieth annual meeting of the Mississippi Valley Medical Association will be held at Hot Springs, Ark., November 20, 21, 22, and 23. The Hotel Eastman will be the place of meeting. The railroad rates will be low; everybody is invited, and a good time promised. The Chairman of the Committee of Arrangements is Dr. Thos. E. Holland, Hot Springs.

---

The Honorable Clark Bell, of the New York bar, doesn't believe in railway spine. He read a paper on the subject before the late meeting of the Railway Surgeons in Galveston, in which he made the remarkable statement that railway spine is "absolutely unknown among savages." So far as we know, Mr. Bell is the first to have made this important observation. But any first-course medical student could have told Mr. Bell that.

---

The seventh annual session of the Southern Surgical and Gynecological Association will be held in Charleston, November 13th, 14th, and 15th, and promises to be the most successful in the history of the organization. Papers will be presented by the leading surgeons and gynecologists of the South. The medical profession is cordially invited to attend. Dr. Cornelius Kollock of Cheraw, S. C., is President; Dr. W. E. B. Davis, Birmingham, Ala., Secretary.

---

We desire to thank our esteemed contemporary, the *North American Medical Review* (Kansas City), for the following comment:

"The Atlanta Medical and Surgical Journal, at the age of forty, in the meridian of life, has grown stronger and better.

Those wishing to keep acquainted with professional thought in the South should take it. It is no less than an excellent magazine."

Our kind and red-headed exchange will please consider the compliment reciprocated, barring the question of age.

---

AT the late meeting of the American Academy of Medicine, the following officers were elected for the ensuing year: President, J. McF. Gaston, of Atlanta, Ga. Vice-Presidents, Rufus P. Lincoln, of New York City; William T. Smith, of Hanover, N. H.; Helen C. Putnam, of Providence, R. I.; Victor C. Vaughan, of Ann Arbor, Mich. Secretary and Treasurer, Charles McIntire of Easton, Pa. Assistant Secretary, Edgar Moore Green, of Easton, Pa. Chairman of Committee of Arrangements, C. C. Bombaugh, of Baltimore, Md. The next meeting will be held in Baltimore, in conjunction with the American Medical Association.

---

THE bronze statue of Dr. J. Marion Sims was unveiled in Bryant Park, New York, October 20th. After addresses by Dr. Geo. F. Shrady and Dr. Paul F. Mundé, the statue was formally presented to the city and accepted by Mayor Gilroy. The erection of this statue is the successful culmination of a movement which began soon after the death of Dr. Sims in 1883. Special praise is due our esteemed friend, the *Medical Record*, for its active work for this object. Contributions were received from the medical profession in this and other countries. There is no more honorable name in American medicine than that of Dr. Sims, and no one could deserve more than he a memorial of this sort. The Woman's Hospital, in the same city, is also his monument.

---

AMONG our advertisers who had exhibits at the Tri-State Meeting were: *The Times and Register*, Medical Journal, Philadelphia; Tarrant & Co., Pharmaceutical Preparations, New York; Doliber-Goodale Co., Mellin's Food, Boston; John B. Daniel, Surgical Instruments, Atlanta; Maltine Manufacturing Co., Infant Food and Dietetic Preparations, New York; Parke, Davis & Co., Pharmaceutical Preparations, Detroit; Sharpe & Dohme, Pharmaceutical Preparations, New York; D. Appleton & Co., Medical Books and Journal, New York; Frederick Stearns & Co., Pharmaceutical Preparations, Detroit; W. B. Saunders, Medical Publications, Philadelphia.

